# STAT5 Is Necessary for the Metabolic Switch Induced by IL-2 in Cervical Cancer Cell Line SiHa

**DOI:** 10.3390/ijms25136835

**Published:** 2024-06-21

**Authors:** Arturo Valle-Mendiola, Leticia Rocha-Zavaleta, Vilma Maldonado-Lagunas, Diego Morelos-Laguna, Adriana Gutiérrez-Hoya, Benny Weiss-Steider, Isabel Soto-Cruz

**Affiliations:** 1 Laboratorio de Oncología Molecular, Unidad de Investigación en Diferenciación Celular y Cáncer, FES Zaragoza, Universidad Nacional Autónoma de México, Batalla 5 de Mayo s/n Col. Ejército de Oriente, Mexico City 09230, Mexico; arturo.valle@zaragoza.unam.mx (A.V.-M.); diego5morelos@gmail.com (D.M.-L.); adrianagh85@hotmail.com (A.G.-H.); bennyweiss@hotmail.com (B.W.-S.); 2 Departamento de Biología Molecular y Biotecnología, Instituto de Investigaciones Biomédicas, Universidad Nacional Autónoma de México, Mexico City 04510, Mexico; lrochaz@biomedicas.unam.mx; 3 Laboratorio de Epigenética, Instituto Nacional de Medicina Genómica (INMEGEN), Periférico Sur no. 4809, Col. Arenal Tepepan, Tlalpan, Mexico City 14610, Mexico; vmaldonado@inmegen.gob.mx; 4 Cátedra CONAHCYT, FES Zaragoza, Universidad Nacional Autónoma de México, Mexico City 68020, Mexico

**Keywords:** IL-2, STAT5, metabolic switch, lactate, NAD+/NADH ratio, cervical cancer

## Abstract

The tumor cells reprogram their metabolism to cover their high bioenergetic demands for maintaining uncontrolled growth. This response can be mediated by cytokines such as IL-2, which binds to its receptor and activates the JAK/STAT pathway. Some reports show a correlation between the JAK/STAT pathway and cellular metabolism, since the constitutive activation of STAT proteins promotes glycolysis through the transcriptional activation of genes related to energetic metabolism. However, the role of STAT proteins in the metabolic switch induced by cytokines in cervical cancer remains poorly understood. In this study, we analyzed the effect of IL-2 on the metabolic switch and the role of STAT5 in this response. Our results show that IL-2 induces cervical cancer cell proliferation and the tyrosine phosphorylation of STAT5. Also, it induces an increase in lactate secretion and the ratio of NAD+/NADH, which suggest a metabolic reprogramming of their metabolism. When STAT5 was silenced, the lactate secretion and the NAD+/NADH ratio decreased. Also, the expression of HIF1α and GLUT1 decreased. These results indicate that STAT5 regulates IL-2-induced cell proliferation and the metabolic shift to aerobic glycolysis by regulating genes related to energy metabolism. Our results suggest that STAT proteins modulate the metabolic switch in cervical cancer cells to attend to their high demand of energy required for cell growth and proliferation.

## 1. Introduction

Cancer is a multifactorial disease initiated by the transformation of the cells, which proliferate abnormally and uncontrollably. These malignant cells show some particular characteristics: sustained proliferative signals, evasion of growth suppressors, genomic instability and mutations, evasion of apoptosis, invasive capacity and metastasis, and metabolism deregulation [1]. The tumor cells reprogram their metabolism to cover their high bioenergetic demands for maintaining high and uncontrolled growth. For example, normal differentiated cells use oxidative phosphorylation to generate the necessary energy and biomass for the normal cellular process and to generate antioxidants that are used to counterbalance reactive oxygen species created during rapid proliferation. To offset potential deficits in energy production, cancer cells typically increase their uptake of extracellular glucose; however, most cancer cells show fundamental changes in their metabolism and depend on aerobic glycolysis. This change is known as the Warburg effect [2,3,4,5,6]. It has been reported that energetic metabolism, in particular glucose metabolism, is connected with the control of cell growth through the activation and silencing of specific tumor genes, leading to uncontrolled cell proliferation, the arrest of the cell cycle and the aging of the cell [7,8]. 

The function and structure of the IL-2 receptor (IL-2R) have been well characterized in lymphocytes, and its function as a necessary signal for cell proliferation has been established [9]. Intracellular signaling initiates by ligand-induced heterodimerization of the IL-2R beta and gamma chains, which results in the activation of multiple intracellular kinases, including members of the JAK family. The IL-2R is expressed in non-hematopoietic cells, especially in different tumor cells [10,11,12]. The IL-2R is associated with the Janus kinases 1 and 3 (JAK1 and JAK3), which are tyrosine kinases that undergo rapid tyrosine phosphorylation upon ligand binding and play a critical signaling function downstream of cytokine receptors [13,14]. JAKs activate Signal Transducer and Activator of Transcription (STAT) proteins to convey the proliferation signal into the cell nucleus [15]. Upon ligand binding to the receptor, STAT proteins become phosphorylated in tyrosine, dimerize, translocate to the nucleus, and regulate the expression of genes that modulates cellular functions. Increasing evidence suggests that STAT signaling may be involved in regulating cellular metabolism [16]. 

STAT5 alludes to two highly related proteins, STAT5A and STAT5B; these proteins share an amino acid sequence similarity of up to 90%. The targets for these transcription factors can be redundant and non-redundant activities; for example, STAT5A and STAT5B show redundant roles in proliferation and apoptosis; in contrast, STAT5A is associated with the regulation of neural development, and STAT5B regulates genes implicated in T cell development and function [17]. Since the differences in the DNA binding domain, C-terminal and N-terminal are minor and due to redundancy roles in proliferation and apoptosis, we will refer to STAT5 in this study.

STAT5 is a novel molecule related to the metabolic switch, mainly when it is phosphorylated. For example, the mitochondrial localization of STAT5 tyrosine phosphorylated was observed in leukemic T cells (these cells express a constitutively activated STAT5), and the presence of STAT5 is increased by IL-2 stimulation; in contrast, the cells without IL-2 did not show STAT5 in the mitochondria [16]. The mitochondrial localization of STAT5 suggests that it may be involved in mitochondrial gene regulation. Also, it coincides with the metabolic shift to aerobic glycolysis, which has been observed in T cells and leukemias stimulated with cytokines. Glucose uptake was enhanced in the hematopoietic stem cells (HSC) expressing STAT5, and HIF-2α is required for the upregulation of genes associated with glucose metabolism; in T cells, it also has been observed that STAT5 mediated the glucose uptake [18]. Both isoforms of HIF, HIF-1α and HIF-2α, regulate the expression of numerous common genes, while HIF-1α induces preferentially genes of the glycolytic pathway [19,20]. 

However, the role of STAT proteins in the metabolic switch of cervical cancer cells is poorly understood. Here, we determined the role of IL-2 in the induction of aerobic glycolysis by measuring lactate secretion, the NAD+/NADH ratio and the role of STAT5 in these metabolic responses in the cervical cancer cell line SiHa.

## 2. Results

### 2.1. Cervical Cancer Cell Lines Express the Different Subunits of the IL-2 Receptor and Proliferate in Response to IL-2

**Figure 1:**
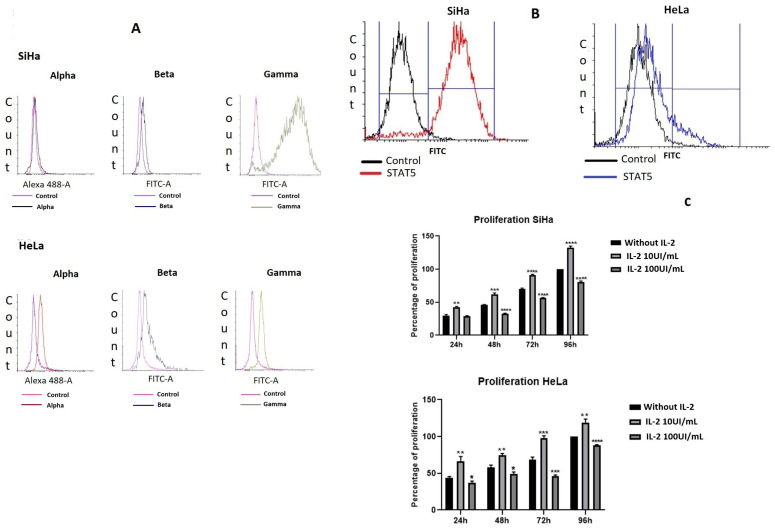
**The IL-2 receptor is present in cervical cancer cells, and cell proliferation increases in response to IL-2.** The expression of the IL-2 receptor was evaluated in SiHa and HeLa cell lines by flow cytometry (**A**). Also, the presence of STAT5 was evaluated in both cell lines by flow cytometry (**B**). Cells were treated with 10 or 100 IU/mL of IL-2 for different times, and their proliferation was evaluated by the violet crystal colorimetric assay (**C**). * *p* < 0.05, ** *p* < 0.01, *** *p* < 0.001, **** *p* < 0.0001.

We determined the presence of the three subunits of the IL-2R (Figure 1A). The most abundant subunit in the SiHa cell line was IL-2Rγ, which was in 85.52% of the cell population, IL-2Rα was not detectable, and IL-2Rβ was in only 1.5%. On the contrary, the HeLa cell line expressed alpha (8.2%), beta (11.06%) and gamma (25.93%) subunits. We also measured the presence of the transcription factor STAT5 by flow cytometry (Figure 1B); the presence of STAT5 in SiHa cells was evident since 88.62% of the cell population expressed this protein; in contrast, in HeLa cells, only 17.17% of the cell population expressed this molecule. Next, we evaluated the effect of IL-2 on cell proliferation (Figure 1C) in both cell lines. When SiHa cells were treated with 10 UI/mL of IL-2 for different periods, their proliferation increased compared to the negative control (24 h by 13.14%, 48 h by 16.14%, 72 h by 20.6%, and 96 h by 32.2%). Nevertheless, the treatment with 100 UI/mL decreased cell proliferation (48 h by 13.17%; 72 h by 13.9%, and 96 h by 19.13%). The same effect was observed for the HeLa cell line, IL-2 enhanced cell proliferation when treated with 10 UI/mL (24 h by 23%, 48 h by 16.84%, 72 h by 28.98%, and 96 h by 19.17%). On the contrary, 100 UI/mL of IL-2 decreased cell proliferation (24 h by 6.28%, 48 h by 8.8%, 72 h by 22.51%, and 96 h by 12.2%).

### 2.2. IL-2 Induced Tyrosine Phosphorylation of STAT5 

**Figure 2:**
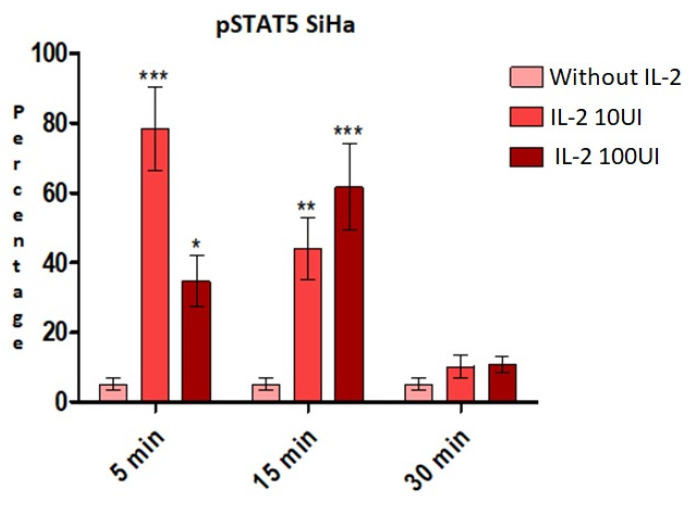
**STAT5 tyrosine phosphorylation increases in response to IL-2.** SiHa cells were treated with 10 IU/mL or 100 IU/mL of IL-2 for the indicated time points and tyrosine phosphorylation of STAT5 was evaluated by flow cytometry. The proportion of cells showing phosphorylated STAT5 is indicated as percentage. * *p* < 0.05, ** *p* < 0.01 and *** *p* < 0.001.

We determined if IL-2 induced the activation of STAT5 in the cervical cell line SiHa, since these cells express a large amount of STAT5 compared to HeLa cells. We incubated the cell line SiHa with 10 or 100 UI/mL of IL-2 at different times to induce tyrosine phosphorylation of this transcription factor (Figure 2). After five minutes of treatment with 10 UI/mL of IL-2, an increase of 73.2% in tyrosine phosphorylation was observed compared to the negative control. After 15 min, cells treated with 10 UI/mL of IL-2 showed an increase of 38.8%. On the contrary, treatment with 100 UI/mL of IL-2 for 5 min only increased tyrosine phosphorylation 29.54%, and the cells treated with 100 UI/mL of IL-2 for 15 min had an increase of 56.55%. Finally, after 30 min of treatment, the phosphorylation difference was not significant (5% with 10 UI/mL and 5.6% with 100 UI/mL).

### 2.3. IL-2 Modulates Lactate Secretion in a Dose-Dependent Manner in SiHa Cells

**Figure 3:**
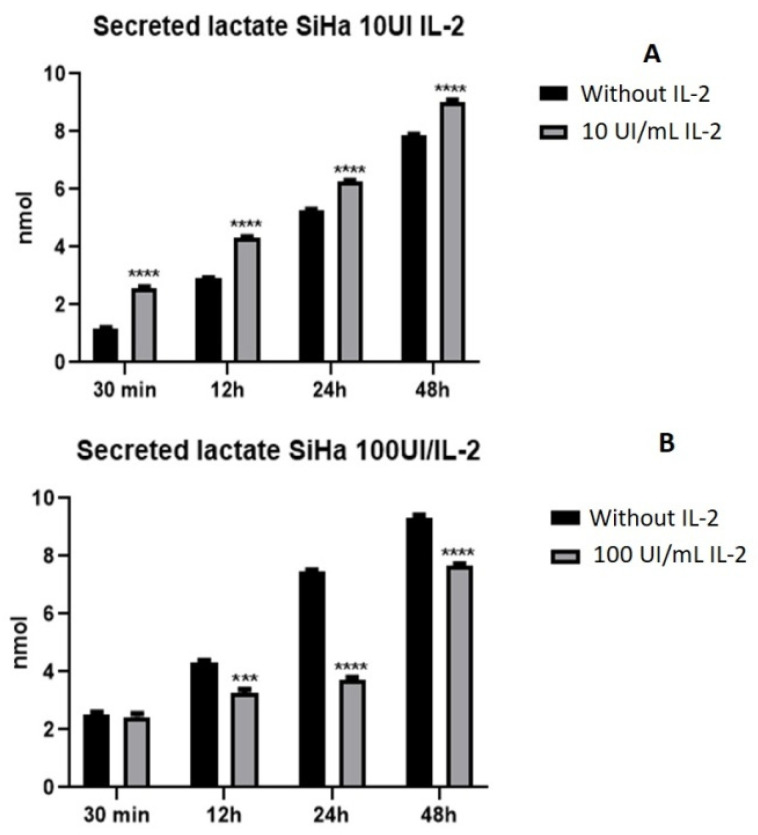
**IL-2 modulates lactate secretion in the cervical cancer cell line SiHa.** Cells were treated with 10 IU/mL (**A**) or 100 IU/mL (**B**) of IL-2 for different times and lactate secretion was evaluated using a commercial Lactate Assay Kit. The absorbance was measured at 570 nm. *** *p* < 0.001; **** *p* < 0.0001.

High lactate secretion is one of the main characteristics of tumor cells; therefore, we evaluated the effect of IL-2 on lactate secretion. The results showed that the IL-2 had a different effect on lactate secretion depending on the dose. When cells were treated with 10 UI/mL of IL-2, lactate secretion increased as early as 30 min up to 48 h (Figure 3A). On the contrary, by stimulating cells with 100 UI/mL, the production of lactate decreased (Figure 3B).

### 2.4. IL-2 Induced an Increase in the NAD+/NADH Ratio in SiHa Cells

**Figure 4:**
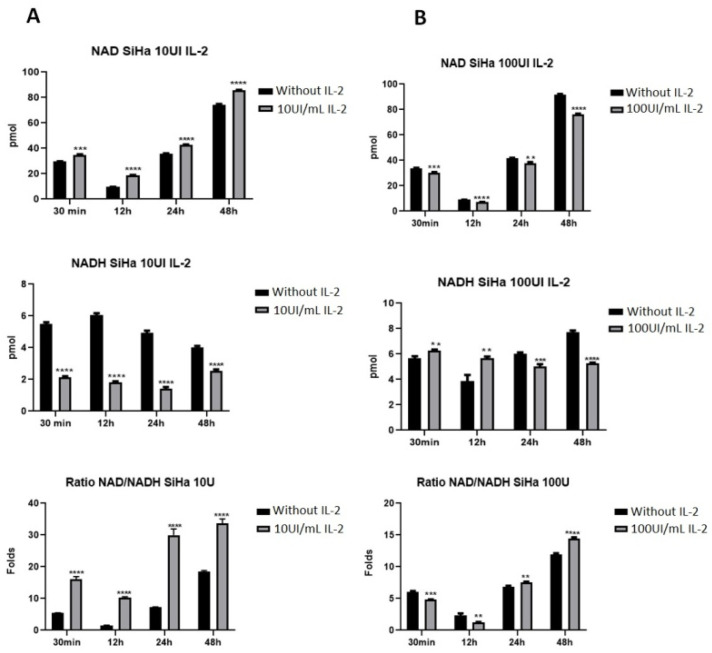
**IL-2 increases the NAD+/NADH ratio in the cervical cell line SiHa.** Cells were treated with 10 IU/mL (**A**) or 100 IU/mL (**B**) of IL-2 for different times, and NAD+ or NADH production was evaluated using a commercial NAD+/NADH Quantitation Kit. The absorbance was measured at 450 nm. ** *p* < 0.01; *** *p* < 0.001; **** *p* < 0.0001.

The pyruvate produced during the aerobic glycolysis is converted to lactate by the lactate dehydrogenase, and the NADH is oxidized to NAD+; therefore, we measured the quantity of NAD+ and NADH. The results showed that NAD+ is more abundant than NADH after treatment with 10 UI/mL of IL-2 (Figure 4A). There was an increase in NAD+ during different treatment times contrasting with the decrease in NADH. It is evident that the NAD+/NADH ratio increased. When the cells were treated with100 UI/mL (Figure 4B), there was a decrease in NAD+ and NADH concentration; also, the ratio of NAD+/NADH decreased compared to the ratio of cells treated with 10 UI/mL of IL-2. 

### 2.5. IL-2 Modified the Expression of Genes Related to Energy Metabolism

**Figure 5:**
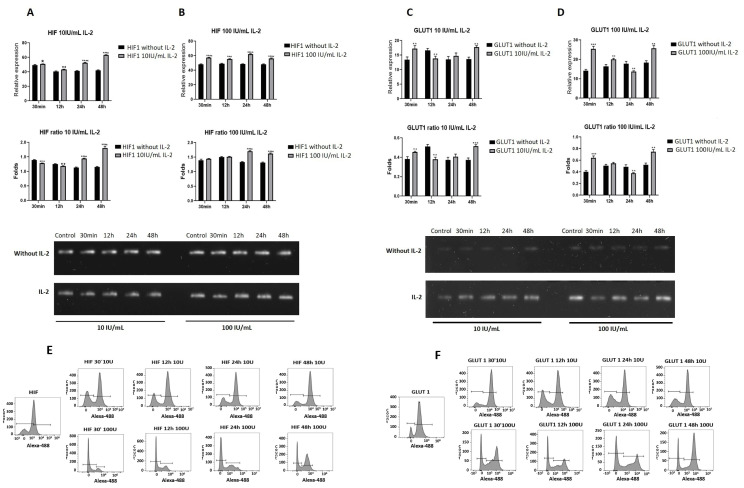
**Genes related to energy metabolism are modified in response to IL-2 in the cervical cell line SiHa.** The expression of HIF and GLUT1 was evaluated by RT-PCR. Cells were treated with 10 IU/mL (**A**,**C**) or 100 IU/mL (**B**,**D**) of IL-2 for different times. Protein levels were evaluated by flow cytometry (**E**,**F**). Densitometric analysis for the time-course of gene expression is shown for each gene as relative expression. * *p* < 0.05; ** *p* < 0.01; *** *p* < 0.001; **** *p* < 0.0001. The following primers were used: HIF1α Forward CCA GAA GAA CTT TTA GGC CGC, Reverse TGT CCT GTG GTG ACT TGT CC; GLUT1 Forward GGA CAG GCT CAA AGA GGT TAT G, Reverse AGG AGG TGG GTG GAG TTA AT. The statistical analysis for three different flow cytometry assays are shown in Supplementary Figure S1.

Because some studies have demonstrated that IL-2 regulates the expression of genes related to energetic metabolism, we determined the effect of IL-2 on the expression of GLUT1 and HIF1. SiHa cells were treated with 10 and 100 UI/mL of IL-2 for 30 min and 12, 24, and 48 h. The results showed that the treatment with 10 or 100 UI/mL of IL-2 increased HIF1a expression (Figure 5A,B). On the contrary, the effect on GLUT1 expression is not the same. IL-2 induced an increase in GLUT1 as early as 30 min; then, it decreased at 12 and 24 h and increased again at 48 h to the same levels as 30 min (Figure 5C,D).

Next, we determined the effect of IL-2 on the proteins GLUT1 and HIF1 (Figure 5E,F); the SiHa cells were treated at the same time and concentrations as those for mRNA analysis. The treatment with 10 UI/mL of IL-2 induced an increase in HIF1 protein at all times (30 min, 67.92%; 12 h, 65.62%; 24 h, 67.92%; and 48 h, 76.42%) compared to the control (46.32%). In the case of 100 UI/mL of IL-2, the effect on HIF1 is the opposite, inducing a decrease in HIF1 protein from 30 min to 24 h (30 min, 16.62%; 12 h, 20.32%; and 24 h, 18.02%) and at 48 h increases (54.82%) compared to the control (46.32%). For GLUT1 protein, the treatment with 10 UI/mL, at 30 min (78.72%), the amount of the protein was similar to the control (80.92%), decreased at 12 and 24 h (58.42 and 61.52, respectively) and increased again at 48 h. When the cells were treated with 100 UI/mL, the level of GLUT1 protein decreased from 30 min to 24 h (30 min, 50.2%; 12 h, 52%; and for 24 h, 62.82%) with a recovery at 48 h (70.62%). Statistical analysis for these data is shown in Supplementary Figure S1.

### 2.6. STAT5 Silencing Decreases Cell Proliferation

**Figure 6:**
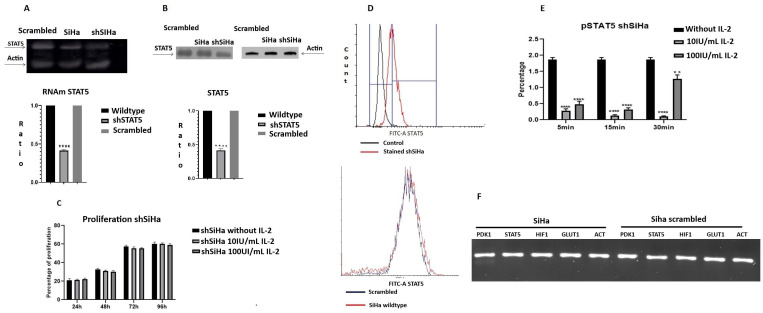
**Effect of STAT5 silencing on cell proliferation and STAT5 phosphorylation.** STAT5 was silenced using an shRNA and the effect of the knockdown was evaluated by RT-PCR (**A**), Western blot (**B**) and flow cytometry (**D**). Also, the phosphorylated form of STAT5 in response to IL-2 treatment was evaluated by flow cytometry, and the proportion of cells showing phosphorylated STAT5 is indicated as percentage (**E**). Siha cell line was transfected with RNA scrambled, and we evaluated the effect in the expression of different genes (**F**). Cells were incubated in the presence of IL-2 for different times, and proliferation was evaluated using the violet crystal colorimetric assay (**C**). ** *p* < 0.01; **** *p* < 0.0001.

To analyze the role of STAT5 on cervical cancer cells metabolism, the STAT5 gene was silenced in the cell line Siha using an shRNA and its corresponding scrambled control; a stable subclone was obtained (shSiHa) (Figure 6). The expression of STAT5 mRNA decreased by 58% (Figure 6A), and the presence of STAT5 protein decreased 59% (Figure 6B). The absence of STAT5 protein affected cell proliferation, since the cells grew slowly in comparison to wild-type cells. However, it is interesting to note that proliferation of the shSiHa subclone was not modified either by 10 UI/mL or 100 UI/mL of IL-2 (Figure 6C). Next, we determined the amount of STAT5 protein and its phosphorylation. As expected, the presence of STAT5 decreased (31.21%) in comparison with SiHa wild-type cells (88.62%, Figure 6D). We incubated the subclone shSiHa in the presence of 10 or 100 UI/mL of IL-2 for different times to induce tyrosine phosphorylation of STAT5 (Figure 6E); after five minutes, there is a dramatic decrease in STAT5 phosphorylation both in the control (1.87%) and in the treatment with 10 UI/mL (0.28%) or 100 UI/mL (0.48%) of IL-2. After 15 min, the phosphorylation decreased with both treatments compared to the control.

### 2.7. STAT5 Modulates the Production of Lactate and the NAD+/NADH Ratio in SiHa Cells

**Figure 7:**
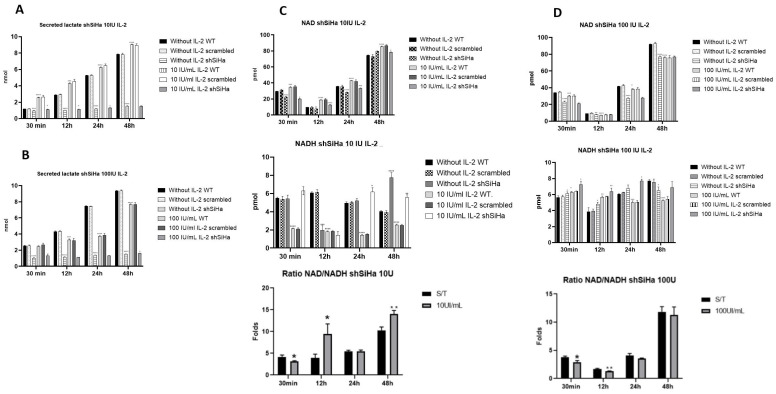
**The production of lactate and the NAD+/NADH ratio decreased after STAT5 knockdown.** The shSiHa subclone was incubated in the presence of IL-2 for different times, and the lactate secretion (**A**,**B**) and NAD+/NADH (**C**,**D**) ratio were evaluated using commercial kits as indicated. * *p* < 0.05; ** *p* < 0.01; *** *p* < 0.001; **** *p* < 0.0001.

Next, we evaluated the effect of STAT5 silencing on lactate secretion (Figure 7). The results showed that the production of lactate decreases approximately ten times when STAT5 is silenced compared to wild-type cells; the effect of both treatments of IL-2 is similar in the shSiHa subclone (Figure 7A,B). Also, we determined the amount of NAD+ and NADH and the ratio present in the shSiHa clone (Figure 7). The results showed that the absence of STAT5 affected the production of these metabolites since the production of NAD+ decreased; there was a slight increase in the production of NADH after treatment with 10 UI/mL of IL-2 compared to wild-type cells and the ratio of NAD+/NADH decreased (Figure 7C). The NAD+/NADH ratio increased when the cells were treated with 100 UI/mL (Figure 7D); there was a decrease in NAD+ and NADH concentration as well. Also, the ratio of NAD+/NADH decreased compared to the ratio of cells treated with 10 UI/mL of IL-2.

### 2.8. STAT5 Regulates the Expression of Genes Related to the Metabolic Switch in Cervical Cancer Cells

**Figure 8:**
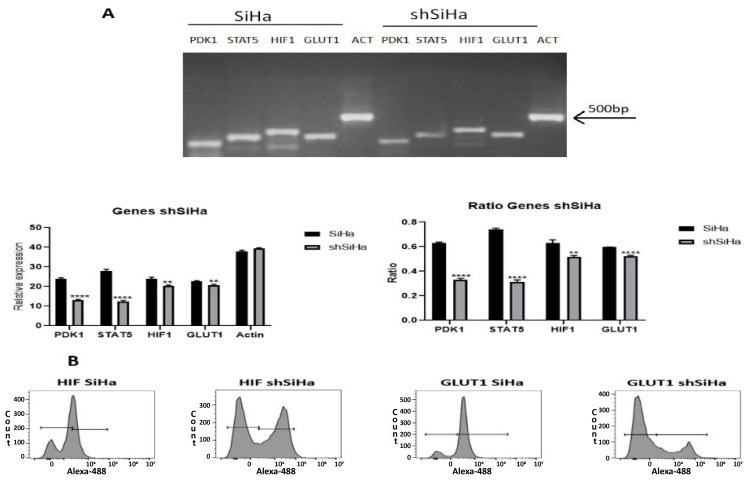
STAT5 regulates the expression of RNA (**A**) or proteins (**B**) of genes related to the metabolic switch in the cervical cancer cell line SiHa. In the shSiHa subclone, the expression of RNA of genes (**A**) related to energy metabolism was evaluated by RT-PCR. The expression of actin was used as an internal control for RT-PCR. Densitometric analysis for gene expression is shown for each gene as relative expression. ** *p* < 0.01; **** *p* < 0.0001. The following primers were used: HIF1a Forward GACAAGCCACCTGAGGAGAG, Reverse GTGGCAACTGATGAGCAAGC; GLUT1 Forward GAACTCTTCAGCCAGGGTCC, Reverse TCACACTTGGGAATCAGCCC; PDK1 Forward AAGTTCATGTCACGCTGGGT, Reverse GCATCTGTCCCGTAACCCTC; STAT5 Forward GGTGAAGGCCACCATCATCA, Reverse GTACTCCATCACGCAGCAGT. To complement these results, we analyzed the presence of HIF-1α and GLUT1 proteins by flow cytometry (**B**).

We analyzed the effect of STAT5 knockdown on the expression of PDK1, HIF-1α and GLUT-1 (Figure 8). As expected, the expression of STAT5 decreased (55.78%). When STAT5 was silenced, the expression of PDK (45%), HIF-1α (14.78%) and GLUT-1 (8.12%) decreased. The ratio of all genes decreased: for STAT5, 0.74 vs. 0.31; for PDK1, 0.63 vs. 0.33; for HIF1, 0.63 vs. 0.51; and for GLUT1, 0.59 vs. 0.52.

Then, we evaluated the presence of the proteins GLUT-1 and HIF-1α (Figure 8B) after STAT5 knockdown using flow cytometry. As expected, GLUT-1 decreased when STAT5 was silenced (7.96% vs. 62.46% in the control). Unexpectedly, in the case of HIF-1α, the protein increased (36.3%, mean fluorescence intensity 14,288 vs. 30.3%, mean fluorescence intensity 4348).

## 3. Discussion

IL-2R is expressed on non-hematopoietic cells, especially on several types of tumor cells like melanoma [10], human squamous cell carcinoma of the head and neck (SCCHN) and keratinocytes [11], epithelial cells [12], and cervical cancer cell lines (HeLa and INBL, HPV-18+) [21]. In this study, we determined the presence of IL-2R (α, β and γ chains) in the cervical cancer cell lines SiHa (HPV-16+) and HeLa (HPV-18+) (Figure 1A). As expected, the cell line HeLa expressed all three subunits of IL-2R; these data are consistent with those reported by Lagunas-Cruz [21]. Surprisingly, the more abundant subunit in SiHa cells is IL-2Rγ (85.52%); on the contrary, the beta subunit is barely present (1.5%), and the alpha subunit is not detectable. One report shows the presence of mRNA for the three subunits of IL-2R in the cervical cancer cell line SiHa [22], and the major subunit is IL-2Rγ. However, they do not show the presence of the protein. 

Previously, we demonstrated that cervical cancer cell lines CALO and INBL (HPV18+) expressed the transcription factor STAT5 [23,24] and are phosphorylated in response to IL-2. Thus, we determined the presence of STAT5 in both cervical cell lines SiHa (HPV16+) and HeLa (HPV18+) by flow cytometry (Figure 1B); as expected, STAT5 was expressed in both cell lines, but SiHa cells had a higher STAT5 (88.62%) content than HeLa cells (17.17%). STAT5 contributes to the pathology of various cancers, like breast, colorectal, lung, prostate, liver, cervical, and hematological malignancies [23,25]. STAT5 plays a pivotal role in cervical oncogenesis; for example, pSTAT5 is important for the amplification of the viral genome [26]. The transcription factor KLF13 (Krüppel-like factor 13) is vital for ATM activity and for STAT5 activation [27], and some studies showed that pSTAT5 plays a major role in cervical cancer pathogenesis [23,28]. We have previously demonstrated that low doses of IL-2 (10 IU/mL) increased the proliferation of cervical cancer cells [23,24], and with high doses of IL-2 (100 IU/mL), the proliferation decreased [21]; as expected, 10 IU/mL of IL-2 induced a significant increase in proliferation on both cell lines (Figure 1C), and 100 UI/mL decreased proliferation, which indicates that this response to IL-2 is maintained regardless of the type of HPV. 

Since IL-2 induces a response in cervical cancer cells, we evaluated the tyrosine phosphorylation of STAT5 in the cell line SiHa due to its higher expression of STAT5. Previously, we reported that the peak of phosphorylation in cervical cancer cell lines treated with 10 UI/mL was 35 min, and by stimulating with 100 UI/mL, this phosphorylation decreased [23]. In this report, we showed that the peak of STAT5 phosphorylation is at 5 min in response to 10 UI/mL (73.2%, Figure 2) in cervical cancer cell line SiHa. Contrary to the data reported for cell lines HPV-18+, when we treated the cells with 100 UI/mL of IL-2, we found an increase in tyrosine phosphorylation, albeit to a lesser extent (29.54%) than the phosphorylation induced by small doses of IL-2. This different behavior in the cervical cancer cell line SiHa could be due to the presence of HPV16 compared to the cell lines used in previous reports, which are HPV18+. One previous report has shown STAT5 phosphorylation in cell lines and CIN lesions of an increasing grade of cervical cancer [28]; however, the phosphorylation level in the SiHa cell line is low compared to our results. Sobti et al. demonstrated the expression of mRNA and the presence of STAT5 protein in fresh cervical cancer specimens [29], and Chen et al. only showed immunohistochemical studies but not the tyrosine phosphorylation of STAT5 [30]. Despite these differences in phosphorylation response, IL-2 induced cell proliferation in a dose-dependent manner similar to previously reported data [21,23]. The cervical cancer cell lines HeLa and SiHa increased their proliferation with 10 UI/mL of IL-2; however, when treated with 100 UI/mL, proliferation decreased. This response indicates that IL-2 has the same effect, at least at the level of proliferation, regardless of HPV type.

On the other hand, the secreted lactate induced by 10 IU of IL-2 in cervical cancer cell line SiHa increased (Figure 3A), in contrast to the decrease in lactate production induced by 100 IU/mL (Figure 3B) of IL-2 showing a dose-dependent effect, which was similar to that obtained in cell proliferation. It is remarkable that stimulation with 10 IU/mL of IL-2 increases lactate secretion; this phenomenon is likely related to STAT5 activation (Figure 2). The increase in proliferation causes an increase in lactate secretion, and the decrease induced by 100 IU/mL is correlated with the lowest STAT5 activation and with the reduction in cell proliferation. This phenomenon shows that the benefit of a deregulated metabolism is enhanced by the presence of metabolites that fuel anabolic pathways, thus sustaining cell proliferation [31,32]. The secretion of lactate is typical of several types of cancer in addition to cancer cells exposed to low oxygen concentration. Nonetheless, lactate levels are elevated in glycolytic tumors and correlate with cancer aggressiveness and poor survival [33,34,35]; hence, the lactate production and HIF-1 expression increase due to the presence of the oncogenic viral proteins E6 and E7 [36]. Lactate is a mediator causing chronic inflammation [37]. Moreover, this lactate secretion in the tumor microenvironment has as a consequence that the immune system cells are unable to release their lactate. The acidification and lactate in the medium suppress tumor necrosis factor (TNF) secretion and proliferation as well as inhibit the cytokine production of human cytotoxic T lymphocytes (CTLs) [38,39]. However, the lactate effects are not restricted to the above; lactate can inhibit histone deacetylases (HDACs), which results in the hyperacetylation of histones H3 and H4, diminishes DNA compactness and modulates the DNA repair process, enhancing the resistance of cervical carcinoma cells to anticancer treatments [40,41]. Lactate is a multi-faceted molecule, a nutrient, a signaling molecule with hormone-like properties, and a molecule able to affect epigenetics [42,43]. Thus, lactate is a by-product of glycolysis and a central molecule in cancer development and maintenance.

NAD+ is an important enzymatic cofactor in central energy metabolic pathways like glycolysis, β-oxidation and the Krebs cycle [44]. NAD+ and its reduced counterpart NADH are essential coenzymes in redox reactions, and a disproportion in their ratio results in an unregulated cellular metabolism [44,45]. One hallmark of the Warburg effect is a high NAD+/NADH ratio [46,47]; hence, this ratio and the NAD+ concentration are higher in cancer cells. These characteristics are consistent with a higher metabolic flux in malignant cells, and the high ratio of NAD+/NADH sustains aerobic glycolysis [47]. Some reports indicated that STAT5 activation is related to a metabolic shift [16,48]; since we found that the cervical cancer cell line SiHa expressed the IL-2R (Figure 1A) and responded to exogenous IL-2 via STAT5 (Figure 1C and Figure 2), we determined the effect of different IL-2 concentrations (10 and 100 IU/mL) on the NAD+/NADH ratio (Figure 4). A remarkable response observed is the high concentration of NAD+ in all IL-2 treatments; this phenomenon was also noted by Veiga Moreira et al. [46]. The treatment with 10 IU/mL of IL-2 increased NAD+ and the NAD+/NADH ratio, which is consistent with the increase in lactate production and cell proliferation. On the contrary, the treatment with 100 IU/mL of IL-2 induced a decrease in NAD+ concentration and in the NAD+/NADH ratio, which is consistent with cell proliferation and a decrease in lactate production. The transformation of pyruvate to lactate is linked with the conversion of NADH to NAD+, which is mediated by lactate dehydrogenase (LDH). This step is essential as a regeneration of NAD+ during glycolysis; hence, a decrease in lactate production in tumor cells indicates cell growth suppression [49]. Conversely, an increase in lactate production indicates an increase in cell growth. Our results are consistent with the conversion of NADH to NAD+ to increase lactate production and cervical cancer cell proliferation. It has been shown that the use of inhibitors of nicotinamide phosphoribosyltransferase (NAMPT) can reduce NAD+ levels by inhibiting energy metabolism pathways like glycolysis, citric acid cycle and oxidative phosphorylation (OXPHOS), contributing to a decrease in cancer proliferation [45]. High doses of IL-2 may have similar effects to those of nicotinamide inhibitors by decreasing cervical cancer proliferation. On the contrary, high levels of NAD+ improve glycolysis, which enhances cancer cell proliferation and survival; hence, NAD+ can promote tumorigenesis [45]. Thus, low doses of IL-2 induce high levels of NAD+, promoting high proliferation and metabolic flux in cervical cancer cells. 

In the present study, we analyzed the IL-2 effect on the expression of critical genes involved in aerobic glycolysis, such as HIF-1α and GLUT1. Both treatments (10 or 100 IU/mL) triggered an increase in the expression of both genes; these results are consistent with some reports indicating that HIF-1 is expressed in cervical cancer [50], and its mRNA is overexpressed [51]. The IL-2 can induce the gene expression and protein synthesis of HIF-1 through the PI3K/mTOR pathway [52]; our results showed an increase in HIF-1 protein after stimulation with 10 UI/mL of IL-2 and the opposite effect with 100 UI/mL. Also, IL-2 can upregulate GLUT1 [53], and the high expression of GLUT1 and HPV-16 is correlated with poor prognosis [54]. HPV-16 likely uses E7 to promote the expression of HIF-1 and utilizes E6 to activate it; this can induce the expression of GLUT1, HIF-1, and LDHA, among others [55]. Our results indicate that IL-2 regulates the expression of HIF-1α and GLUT1 in a similar way to its effect in cytotoxic T lymphocytes, where HIF-1α controls the expression of some genes like glucose transporters and genes related to metabolism [56].

Recently, several reports indicate that STAT5 participates in metabolism regulation [16,48,57,58,59], and our workgroup has demonstrated that cervical cancer cells express STAT5, and its phosphorylation increased in response to IL-2 [23,24]. Therefore, we used RNAi to knock down STAT5 to analyze its role in metabolism regulation (Figure 8). As expected, the effect of IL-2 on cell proliferation was abolished, which correlated with the decrease in the tyrosine phosphorylation of STAT5, suggesting diminished transcriptional activity. These results indicate the pivotal role of this molecule in cervical cancer cell function. The lactate secretion in the cervical cancer cell subclone shSiHa was dramatically reduced, indicating a possible role of STAT5 in lactate production. Few studies have related lactate production to STAT5; most show indirect evidence, such as an inhibition of aldose reductase or sorbitol dehydrogenase blocking STAT5 activation, which causes a low lactate to pyruvate ratio [60]. JAK2V617F and STAT5 can increase the expression of 6-phosphofructo-2-kinase/fructose-2,6-bisphosphatase 3 (PFKFB3), which is an enzyme required for lactate production [61]. The role of STAT5 in lactate production remains poorly understood and awaits further analysis.

As mentioned above, a high NAD+/NADH ratio is a characteristic of the Warburg effect, and this high ratio is consistent with aerobic glycolysis. Because STAT5 activation is related to the metabolic shift, as expected, STAT5 inhibition reduced the amount of NAD+. NADH remained nearly unchanged, and the ratio of NAD+/NADH decreased dramatically with 10 IU/mL of IL-2. An augment in NAD+ is mediated by NAMPT [60], and this molecule increases with the lesion [62]. NAMPT catalyzes NAD+ synthesis, and its levels and activity determine NAD+ levels [63,64]. High NAMPT expression promotes cell growth, survival, DNA synthesis, mitochondria generation, angiogenesis, and malignant progression [63]. In the case of the cervix, NAMPT is highly expressed in cervical adenocarcinoma [62]. STATs are essential regulators for NAMPT expression; STAT3 activity induces a high expression of NAMPT [65]; in the case of STAT5, this transcription factor can bind to the promoter of NAMPT and induce its expression [66]. In BRAFV600E melanoma cells, the inhibition of STAT5 decreases NAD+ levels via NAMPT inhibition [67], while in cervical cancer cell line SiHa, STAT5 probably regulates the expression of NAMPT because its inhibition reduces NAD+ levels. This phenomenon requires further analysis.

STAT5 silencing inhibits HIF-1 and GLUT1 expression, indicating that this transcription factor regulates the expression of both molecules. The inhibition of STAT5 phosphorylation decreases the expression of HIF-1 and GLUT1 [68]. In bone marrow mesenchymal stem cells, the enhanced expression of HIF-1 is induced at least in part by the JAK2/STAT3/STAT5 pathway [69]; in contrast with mRNA expression, the presence of the protein HIF-1α increased. This result is unexpected, because the mRNA decreases, but protein synthesis increases (Figure 8). This paradoxical result can be explained by the effect of miRNA or heat shock proteins, particularly HSP-90. miRNAs are post-transcriptional regulators of gene expression [70]; for example, miR-155 is capable of negatively regulating HIF-1α during prolonged hypoxia [71], and this miRNA is expressed in cervical cancer [72,73,74], and STAT5 can regulate miR-155 expression [70]. It is possible that STAT5 silencing decreases the amount of miR-155, directly affecting HIF-1α expression, but the molecule that directly stabilizes HIF-1α protein is HSP90 [75,76,77,78] and can regulate HIF-1α protein abundance [79]. On the other hand, it has been demonstrated that HSP90 binds directly to STAT5 [79,80,81] and primarily binds to pSTAT5 [82]. The presence of the HSP90 chaperone is well documented in cervical cancer tissue and cell lines [83,84,85]; one possible explanation for the increase in HIF-1α protein in the subclone shSiHa is that by decreasing the amount of pSTAT5, the HSP90 is released; then, it is available to stabilize HIF-1α, increase protein stability, and in consequence increase the amount and the mean fluorescence intensity.

For GLUT1, STAT5 is required for glucose uptake and surface localization [18,70], and our results show that STAT5 regulates the expression of mRNA and therefore, the protein GLUT-1; Cheng et al. demonstrated that the GLUT-1 mRNA and protein increased with the lesion [50], and this correlates with STAT5 expression [28], because the STAT5 silencing decreases dramatically the presence of the protein. 

Our results show that in cervical cancer cell line SiHa, STAT5 is able, at least in part, to regulate HIF-1 and GLUT1 expression. This behavior indicates that cervical cancer cell lines tend to behave like hematopoietic cells. Altogether, our results indicate that IL-2 increases lactate production and the NAD+/NADH ratio in cervical cancer cells, which is consistent with aerobic glycolysis. Also, STAT5 regulates the expression of genes necessary for this metabolic shift to increase cell proliferation.

## 4. Materials and Methods

### 4.1. Cell Culture Conditions

The HeLa cell line (ATCC-CCL-2) and the SiHa cell line (ATCC-HTB-35) were purchased from the American Type Culture Collection. HPV-associated cervical cancer cell line SiHa was cultured in RPMI 1640 (Microlab, Mexico City, Mexico), and the HeLa cell line was cultured in DMEM (Microlab, Mexico City, Mexico) medium supplemented with 10% fetal calf serum (FCS, Invitrogen, Carlsbad, CA, USA) and were incubated at 37 °C in 5% CO_2_.

### 4.2. Flow Cytometry

We used 0.5 × 10^6^ cells fixed with paraformaldehyde 2% for 20 min and were permeabilized with methanol. The cells were incubated with an antibody anti-STAT5 (Santa Cruz Biotechnology, Santa Cruz, CA, USA) and posteriorly were incubated with the secondary antibody coupled with FITC. To determine pSTAT5, we used an anti-Y694 antibody coupled with PE-eFluor 610 (eBioscience, San Diego, CA, USA); the cells were permeabilized with Cytofix/Cytoperm (BD, San Jose, CA, USA). For the analysis of the IL-2R subunits, we used an anti-CD25 antibody (alpha subunit) coupled with Alexa fluor 488 (BD, San Jose, CA, USA), an anti-beta subunit antibody coupled with FITC (eBioscience, San Diego, CA, USA), anti-gamma subunit antibody (Santa Cruz Biotechnology, Santa Cruz, CA, USA), anti-GLUT-1 (Santa Cruz Biotechnology, Santa Cruz, CA, USA), anti-HIF-1α (Santa Cruz Biotechnology, Santa Cruz, CA, USA) and a secondary antibody coupled with FITC (Santa Cruz Biotechnology, Santa Cruz, CA, USA). The events were detected in a FACSAria II cytometer (BD, San Jose, CA, USA). In the case of GLUT-1 and HIF-1α proteins, the events were detected in a Cytoflex cytometer (Beckam Coulter, Pasadena CA, USA).

### 4.3. Lactate Quantitation

We used the Lactate Assay Kit (Sigma, Saint Louis, MO, USA) under the manufacturer’s conditions. Briefly, we seeded and stimulated 0.5 × 10^6^ cells with 10 or 100 UI/mL of IL-2 for 30 min, 12, 24 and 48 h, and we collected the supernatant that was deproteinized with perchloric acid and neutralized with KOH 1M. We placed 50 µL in 96-well plates, added 50 µL of the master reaction mix, and then incubated and shook the plate for 30 min at room temperature. We measured the absorbance at 570 nm.

### 4.4. Determination of NAD+ and NADH Levels

We used the NAD+/NADH Quantitation Kit (Sigma, Saint Louis, MO, USA) under the manufacturer’s conditions. Briefly, we seeded 0.3 × 10^6^ cells and stimulated with 10 or 100 UI/mL of IL-2 for 30 min, 12, 24, and 48 h. We added 400 µL of NADH/NAD extraction buffer and lysed the cells by freeze/thawing, centrifuged them at 13,000 rpm for 10 min, and then deproteinized the samples with a 10 kDa MWCO spin filter. We placed 50 μL of each sample in duplicate in a 96-well plate; then, we added 100 μL of master reaction mix and incubated the plate for 5 min with gentle shaking. Then, we added 10 μL of the developer to each well, incubated it for 1.5 h at room temperature, and measured the absorbance at 450 nm.

### 4.5. Silencing of STAT5

We designed the following sequences of shRNA for silencing STAT5 (A/B): STAT5-94 CCACCATCACGGACATTAT, STAT5-71 GACTCAGGAGTACTTCATC and the scrambled control hairpin were inserted into a pSIREN plasmid containing the puromycin resistance gene. The cells were transfected using Turbofect (Thermo Scientific, Walthman, MA, USA) under the manufacturer’s specifications. Briefly, we seed 0.6 × 10^6^ cells per well in a 24-well plate 24 h before transfection. We diluted 5 μg of DNA in 400 μL of serum-free culture medium, added 6 μL of Turbofect, mixed immediately and incubated for 20 min at room temperature. We added 400 μL of the mixture in each well, shook it to mix perfectly and incubated at 37 °C, 5% CO_2_. The cells were incubated in a selection medium (puromycin 2 μg/mL) for a stable transfection for 15 days.

### 4.6. Determination of Cell Proliferation

The cell proliferation was measured using the violet crystal technique. Briefly, we seeded 3000 cells/well in a 24-well plate. The cells were cultured for 24, 48, 72 and 96 h. The medium was eliminated, 300 µL of glutaraldehyde 1.1% was added, and then it was incubated for 20 min with gentle shaking at room temperature. 

Once the time had elapsed, the cells were washed twice with water and dried. We added 300 μL of violet crystal 4.3%, incubated it for 20 min with gentle shaking, washed the plate with water to eliminate all the unbound violet crystal, and allowed it to dry at room temperature. We added 300 μL of acetic acid 10%, incubated it for 15 min and measured the absorbance at 570 nm. 

### 4.7. Lysis and Immunoprecipitation

Cells were lysed with ice-cold lysis buffer [1% Triton X-100, 5 mM EDTA, 140 mM NaCl, 50 mM Tris (pH 7.4), 1 mM PMSF, 1 mM NaF, 1% aprotinin, 1 μM leupeptin, 1 μM pepstatin, and 100 μM Na_3_VO_4_] for 15 min. Lysates were clarified by centrifugation at 13,000 rpm at 4 °C for 15 min, and then the supernatants were collected. 

### 4.8. Immunoprecipitation and Immunoblotting

For immunoprecipitation, the cells were lysed as mentioned above. The total protein content of the lysates was determined using the Bio-Rad protein assay (Bio-Rad, Hercules, CA, USA), and 150 μg of protein was incubated with protein A-agarose beads (Invitrogen, Carlsbad, CA, USA) previously coupled with anti-STAT5 antibody (Santa Cruz Biotechnology, Santa Cruz, CA, USA) for 3 h at 4 °C. Immunoprecipitated proteins were washed five times with ice-cold lysis buffer, resolved by 10% SDS-PAGE and transferred to nitrocellulose membranes (Bio-Rad, Hercules, CA, USA). Membranes were blocked in Tris-buffered saline with 0.1% Tween 20 (TBST) and 3% bovine serum albumin for one hour at room temperature. Membranes were analyzed using an anti-STAT5 antibody (Santa Cruz Biotechnology, Santa Cruz, CA, USA) followed by incubation with a horseradish peroxidase (HRP)-conjugated rabbit anti-mouse antibody (Thermo Scientific). Proteins were visualized by using the enhanced chemoluminescence detection system (Super Signal, Pierce, Rockford, IL, USA). The images were obtained with the Chemidoc MP imaging system (Bio-Rad). 

### 4.9. PCR

We seeded 1 × 10^6^ cells, isolated the mRNA using Trizol (Invitrogen, USA) and used 3 µg of RNA for retrotranscription with the enzyme M-MLV-RT (Promega, Madison, WI, USA). The PCR conditions were 94 °C for 5 min, 60 °C for 1 min, 72 °C for 1 min, and 95 °C for 1 min for 30 cycles. The products were visualized in an acrylamide gel. 

### 4.10. Statistical Analysis

All the data were obtained from at least three independent experiments for statistical analysis. The data are presented as the mean ± standard error of the mean. An unpaired Student’s *t*-test (two-tailed) for parametric data was used to compare treatment groups using the GraphPad Prism v8.0.1 statistical package (GraphPad Software, Boston, MA, USA). *p*-values and 95% confidence intervals were calculated. *p* < 0.05 was considered to indicate a statistically significant difference.

## Data Availability

The data presented in this study are available upon reasonable request from the corresponding author.

## Supplementary Materials

The following supporting information can be downloaded at https://www.mdpi.com/article/10.3390/ijms25136835/s1.
