# Supplementary material for: STAT5 Is Necessary for the Metabolic Switch Induced by IL-2 in Cervical Cancer Cell Line SiHa
Source: Int J Mol Sci. 2024 Jun 21;25(13):6835. doi: 10.3390/ijms25136835 (PMC11241652; doi:10.3390/ijms25136835)
Supplement: Supplementary file 1 [file ijms-25-06835-s001.zip › ijms-2980056-supplementary.pdf]

Supplementary Figure S1 to Figure 5

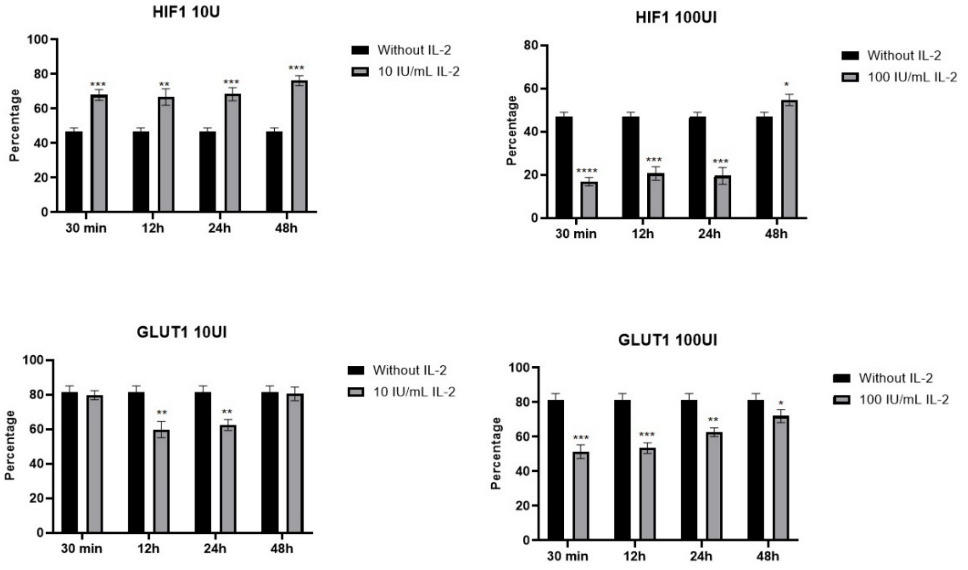

Figure S1. Statistical analysis of flow cytometry assays to determine the presence of HIF1α and GLUT1 proteins in SiHa cells. * $p < 0.05$, ** $p < 0.01$, *** $p < 0.001$, **** $p < 0.0001$.
